# Early and late stage processing abnormalities in autism spectrum disorders: An ERP study

**DOI:** 10.1371/journal.pone.0178542

**Published:** 2017-05-24

**Authors:** Shanshan Wang, Chunjuan Yang, Yijun Liu, Zhi Shao, Todd Jackson

**Affiliations:** 1 Key Laboratory of Cognition and Personality, Southwest University, Chongqing, China; 2 Rehabilitation Center for Children With Autism, Chongqing Ninth People’s Hospital, Chongqing, China; 3 Department of Psychology, University of Macau, Macau, China; McGill University, CANADA

## Abstract

This research assessed event-related potentials (ERPs) elicited during the processing of different kinds of visual stimuli among children with Autism Spectrum Disorder (ASD) (n = 15) and typically developing (TD) children (n = 19). Within a simple visual oddball paradigm, participating children passively viewed fruit and vegetable images that were used as standard stimuli in addition to images of these foods with their usual colors modified to create novel stimuli and cartoon depictions of these images (i.e., “deviant” stimuli). Analyses revealed significant main effect differences between the groups for P100, N100 and P300 components; ASD group children showing longer P100 latencies, weaker N100 amplitudes and larger P300 amplitudes than did the TD group. A Group x Hemisphere interaction also emerged for N400 amplitudes but differences were not significant in simple-effects analyses. Together these results suggested children with ASD may be characterized by lower attention resource allocation and engagement during early stages of processing visual stimuli. However, ERPs in later processing stages suggested children with ASD and TD children have similar neural responses in attending to visual images as stimulus presentations continue.

## Introduction

According to the Diagnostic and Statistical Manual of Mental Disorders–Fifth Edition [1], Autism Spectrum Disorder (ASD) is a severe developmental disorder characterized by deficits in social interaction and communication in addition to stereotyped, repetitive behaviors and restricted interests. People with ASD experience difficulty changing the focus of initial attention, establishing rules, and/or generating solutions because of stereotyped and repetitive behavior that impair flexibility and executive functioning [2,3]. In addition, children with ASD can show indifference or lack of curiosity and motivation regarding their surroundings and changes in context.

Some researchers have identified deficits in sensory information processing modalities among people with ASD [4–9] in addition to modality impairments related to intolerance of change [10]. Deficits in auditory [4–6] and visual [7–9] modalities interfere with novel stimulus processing. As such, these deficits may contribute to associated disturbances in behavior and intolerance of changes [10–12]. Specifically, impaired connectivity within posterior sensory modalities can affect specific attention systems [7,13,14] during exposure to external stimuli, though it is not entirely clear when and how associated perceptual disturbances emerge.

People with ASD can also “over-process” information to successfully differentiate target and novel stimuli while experiencing difficulties processing novel events due to perceptual abnormalities [7]. For them, effective processing requires more effort and resources, though lesions within some brain areas may impede processing goals. Furthermore, persons with ASD can be over-sensitive to changes in visual stimuli [8] and show potential deficits in the automatic processing of changing stimuli [12]. Consequently, even when these individuals exert more effort in processing stimuli accurately, performance may be impaired compared with that of typically developing (TD) people.

In the present study, we attempted to identify neural abnormalities in the course of processing different types of visual stimuli among children with ASD in addition to neural markers of possible compensatory strategies used to reduce possible impairments in visual information processing affected persons can display. Indifference to environments that children with autism show does not necessarily mean that they lack neural responsiveness to identify changes in stimuli or the emergence of novelty compared to TD children. Although people with ASD have difficulties tolerating changes, behaviorally, they can focus on tasks that they like or are required to do [4,7].

During the past decade, researchers have increasingly used various imaging methods to identify neural markers of information-processing deficits and capacities in ASD groups. Event-related potentials (ERPs) have been used to examine neural responses during the time course of stimulus events with higher temporal resolution than alternative strategies such as functional magnetic resonance imaging. In this research, we assessed ERPs elicited during early and later stages of visual stimulus presentations to identify neural correlates of information-processing among children with ASD versus TD children.

Early ERPs, including P100 and N100 components, are indices of initial attention that can be affected by exogenous attributes of stimuli [15] and can affect later stages of stimulus processing [9]. P100 is closely related to selective attention and the consumption of attention resources [16] while N100 is said to reflect attention orientation and/or early engagement [17]; people with ASD may require more attention resources in processing novelty and can display low selectivity for distinct stimulus categories [18]. More specifically, lesions to the lateral extra-striate cortex within this group are associated with abnormal early ERPs vis a vis occipital-parietal areas [19,20]. Such deficits may illustrate comparatively lower consumption of attention resources, low selectivity between stimulus classes, and impairments in early orienting of attention to newly appearing visual stimuli.

In later processing stages, children with ASD may also show significant differences in P300, MMN and N400 components compared to TD children. P300 amplitudes have positive correlations with allocation of attention resources, stimulus recognition, and updating of working memory [21–23]. Longer P300 latencies can reflect higher levels of task difficulty. P300 includes subcomponents that reflect initial detection, reactions to, and evaluations of visual stimuli [24] and has been used as a neural marker for damage of cognitive processing capacities among disabled patients. For instance, low P300 amplitudes and long P300 latencies in patients with Alzheimer’s disease indicate the presence of impairments in cognitive functioning [25]; conversely, shorter latencies of the P3a subcomponent as well as larger P3b subcomponent amplitudes and longer P3b latencies elicited by target stimuli among patients with Parkinson’s disease reflect deficits of automatic processing [26]. Together these studies underscore the utility of P300 as a neural marker for impairments in cognitive processing due to disease-related brain damage.

While N100 and P100 are related to initial orienting and attentional capture, P300 can be used as an index of sustained attention [7,27,28]. Given that groups with ASD and TD groups do not generally show significant differences in the P3b component [29,30], impairments within the former group may not reflect sustained attention. Instead, group differences in P100 and N100 [18–20] suggest that problems related to ASD are more prominent in relation to initial orienting of attention that may be compensated for later stages involving sustained attention, at least when attention performance is comparable between children with ASD and TD children.

Aside from the pattern delineated above, in the present study fixed attributes of visual stimuli were altered to create deviant stimuli and novel stimuli. Within such contexts, mismatch negativity (MMN) might be evoked and is of interest. When incoming stimuli and representations of preceding stimuli in memory differ, MMN can be evoked by the deviant stimuli. MMN reflects an automatic response during such comparisons. When differences between these stimuli are larger, MMN amplitudes may also be larger. For example, in research on schizophrenia, MMN abnormalities suggest impaired automatic processing capacities [31]. MMN effects are often elicited within Oddball paradigms that feature novel stimuli with small probabilities; as such, MMN may be regarded as the specific marker for responsiveness to novel stimuli that has key implications for survival. Other functions reflected in MMN include attention resource allocation and pre-attention changes in the detection process that do not require active participation [32].

Earlier work on visual mismatch responses of children with autism compared to TD children suggested that the former group is hypersensitive to deviance in visual stimulus patterns [32]. Cléry et al proposed that children with autism have general deficits in change detection processing that result in intolerance of changes [8]. However, within the oddball paradigm used in this study, children passively attended to visual stimuli and were not required to generate particular responses upon exposure to novel visual images. Within this context of comparatively low task demands, children with ASD were not expected to differ from TD children on MMN.

Finally, assuming that attention deficits of people with ASD are more prominent in early information processing stages, possible compensatory mechanisms may emerge during later stages of processing. Some research suggests people with autism prefer visual strategies over verbal strategies to facilitate processing. For example, when inner speech is limited, visual-spatial strategies that involve articulator suppression are used more effectively among individuals with ASD [33]. Presumably, then, children with ASD may rely less on semantic strategies than visual-spatial strategies during late stages of processing visual stimuli. Because the N400 component has been the focus of numerous studies on principles of language processing, it may reflect, in part, the degree to which semantic processing, semantic ambiguity, and mental imagery are involved in late stages of visual image processing. Hence, if difference strategies are used to process visual stimuli in late stages, children with ASD and TD children might be expected to differ on the N400 component.

In summary, this study focused on cortical activation during the presentation of standard, deviant, and novel visual images to identify possible neural markers differentiating children from ASD from TD children. As outlined above, we expected these groups to show ERP differences in early stages of visual stimulus processing, with the former group showing longer latencies and/or weaker amplitudes on P100 and N100 components, reflecting possible deficits in orienting, allocation, and capture of initial attention. Conversely, children with ASD and TD children were not expected to differ on ERPs elicited during later stages of stimulus processing reflecting sustained attention or responses to change and novelty (i.e., parietal-occipital P300, central-parietal MMN). Finally, as a reflection of possible differences in preferences for using imagery versus semantic strategies in late processing stages, children with ASD and controls were expected to differ on the N400 component in a manner suggesting the former group relied more heavily on visuospatial than semantic strategies in processing task-related images.

## Methods

### Ethics statement

The study was approved by Southwest University Human Research Institutional Review Board. All parents of participating children and participating children themselves provided written informed consent to engage in the research.

### Participants

Fifteen children with ASD (13 boys, 2 girls) from 3.92 to 8 years of age (M = 5.40 years, SD = 1.22) were assessed. This group included children with autistic disorder or Asperger syndrome who had no co-morbid diagnoses. In addition, 19 gender-matched typically developing (TD) children from 4.0 to 6.5 years of age (M = 5.5 years, SD = 0.78) were included as controls. There was no statistically significant age difference between these groups (p > .05). Furthermore, patterns of significant results did not differ from those summarized below after dropping the youngest or oldest member of the ASD group. Two TD participants were excluded due to too few usable segments of EEG data as the result of recording artifacts (< 38 segments), leaving 17 TD participants.

ASD participants were recruited from a rehabilitation center for children with autism at a local hospital and were diagnosed by a licensed clinician using the Autism Diagnostic Interview (ADI-R; mean Social, 27.3 ± 9; Communication, 19.3 ± 3; RRB, 7.9 ± 3) and Children’s Autism Rating Scale (CARS; mean standard deviation: 37.3 ± 2; threshold for ASD = 30). They also underwent a medical evaluation by a developmental pediatrician to rule out comorbid medical conditions. TD children with evidence of a current or past neurological disorder, serious head injury, or seizures were excluded from the research. All participants in both groups had normal or corrected-to-normal vision.

### Experimental procedure and stimulus materials

In the formal study, all participants were first taught the included fruits and vegetables for 30 minutes followed by a initial recognition task to assess group differences in memory of newly learned stimulus materials. Accuracy rates in initial recognition were virtually identical between groups [ASD group: M = 0.76, SD = 0.06; TD group: M = 0.76, SD = 0.08, t = 0.09, p = 0.93]. This finding suggested there were no obvious group differences in task-related attention, recognition or articulation capacities. Subsequently, a second 30 minute learning phase was undertaken to ensure all children had mastered recognizing each fruit and vegetable to be presented in the formal study. Fruit and vegetable images were then selected at random presented from a book and children were required to recognize them with 100% accuracy before proceeding on to the formal task. All children were able to achieve this criterion, a finding that bolsters support for the absence of group or age-related differences in gross, task-related information processing capacities.

**Fig 1:**
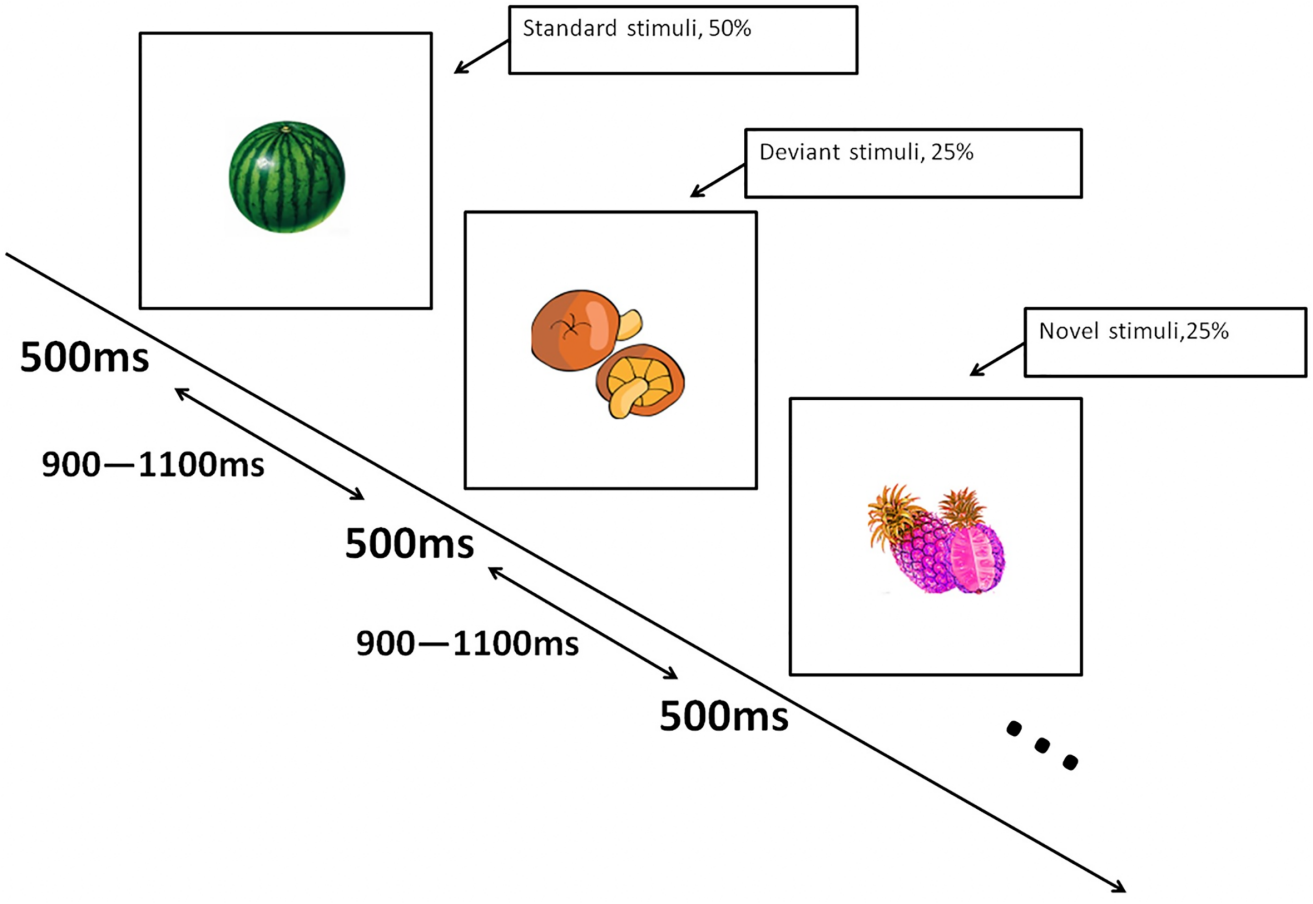
Three stimulus category simple visual Oddball task. There were 16 blocks with 50 trials per block. Participants were instructed to pay attention to all stimuli and to report the names of stimuli they had just viewed during breaks between blocks.

During the formal study task, three categories of fruit and vegetable images were presented as participants passively viewed them. First, 400 pictures of real fruit and vegetables were defined as “Standard stimuli” (50% probability of occurrence). Second, 200 cartoon images of fruit and vegetables were regarded as “Deviant stimuli” (25% probability of occurrence). Third, 200 images of fruit and vegetables with abnormal colors, (e.g., blue watermelon) were “Novel stimuli” (25% probability of occurrence) (Fig 1). All images were 350 x 280 pixels, matched for brightness and contrast, and presented on a white background.

For the main research task, children sat at a viewing distance 70–100 cm from a 14 inch computer screen. Images were presented in 16 blocks of 50 trials each. Each trial comprised one stimulus presented for 500 ms in the center of the screen followed by a 900–1100ms inter-stimulus interval. Blocks were matched for number of standard, deviant, and novel images which were presented in random order. There was a 1–2 minute break after each block during which participants were asked to recall as many different fruits/vegetables as they could via free recall. The purpose of the post-block recall task was to reinforce the importance of participants’ attending to the screen during subsequent stimulus presentations and recall results were not recorded. On average, the task took about 34–51 minutes to complete.

### EEG recordings

Electroencephalography (EEG) data were recorded from 64 scalp sites using tin electrodes mounted in an elastic cap (Brain Products, Munich, Germany) at a 500 Hz sampling rate with a 0.05–100 Hz bandpass filter. Electrodes in the cap were placed according to the International 10–20 system. One additional lead was affixed to the infraorbital of the right eye (as the vertical electrooculogram (VEOG)), and also recorded ERPs of the outer canthi of the right and left eyes [as the horizontal electrooculogram (HEOC)] and the left and right mastoid sites. EEG signals were referenced to left and right mastoid recordings. Electrode impedances were kept below 5 kΩ. In addition, in order to control for ERP effects due to unequal numbers of trials between image types, ERPs of 200 standard stimuli were recorded.

Offline, data were digital bandpass filtered from 0.01 to 16 Hz (24 dB/oct), with an additional notch filter at 50Hz to suppress line activity; data were re-referenced to the average of the left and right mastoids. All data processing steps were performed with the Vision Analyzer 2.1 (Brain Products, Gilching, Germany). Data were segmented into 800 ms duration epochs, including a 200 ms pre-stimulus baseline recording. Trials with EEG voltages that exceeded ±100 μV were excluded from analysis. Additionally, an independent component analysis (ICA) was used to remove ocular artifacts (blinks and saccades), and artifact rejection was performed to exclude effects of muscle or recording artifacts and excessive noise.

### Data analysis

Initially, mean amplitudes and latencies of N100 (40–80 ms) and P100 (80–180 ms) were analyzed. Both components were localized in occipito-parietal sites and included the following EGI channels: P3, P5, PO3, O1 (left) and P4, P6, PO4, O2 (right). As well, P300 (320–560 ms) and MMN (200–350 ms) components were analyzed from central-parietal and occipito-parietal electrodes, respectively. Electrodes for P300 included CP1, CP3, P1, P3 and corresponding electrodes on the right hemisphere. Electrodes for MMN included P3, P5, PO3, O1 and corresponding electrodes on the right hemisphere. N400 (320–560 ms) data from the central-frontal site ERPs included: F3, F1, FC1, FC3 and corresponding electrodes on the right hemisphere.

Group (ASD, TD) × Hemisphere (left, right) × Image Type (deviant, novel, standard) x Electrode repeated measures analyses of variance (ANOVAs) assessed mean latency and amplitude effects for each component of interest. Group and Image Type were between-groups factors while Electrode and Hemisphere were within- participants factors. In main analyses, the p-value for significance (p < 0.01) was set to be more stringent than the conventional p < 0.05 significance level to reduce the risk of Type I errors resulting from multiple analyses of the five ERP components of interest. The significance level was adjusted according to sphericity violations and the Greenhouse-Geisser correction was reported. When significant interactions emerged, data were analyzed with a Bonferroni-correction; student t-tests and one-way ANOVAs were used for within simple effects analyses. Because ASD group differences were study focus, only main effects of Group (ASD vs. TD) and their interactions with other independent measures are presented below. A summary of all other results peripheral to the main research focus can be obtained by contacting the corresponding author. All analyses were performed using SPSS 17.0.

## Results

### P100 component

**Fig 2:**
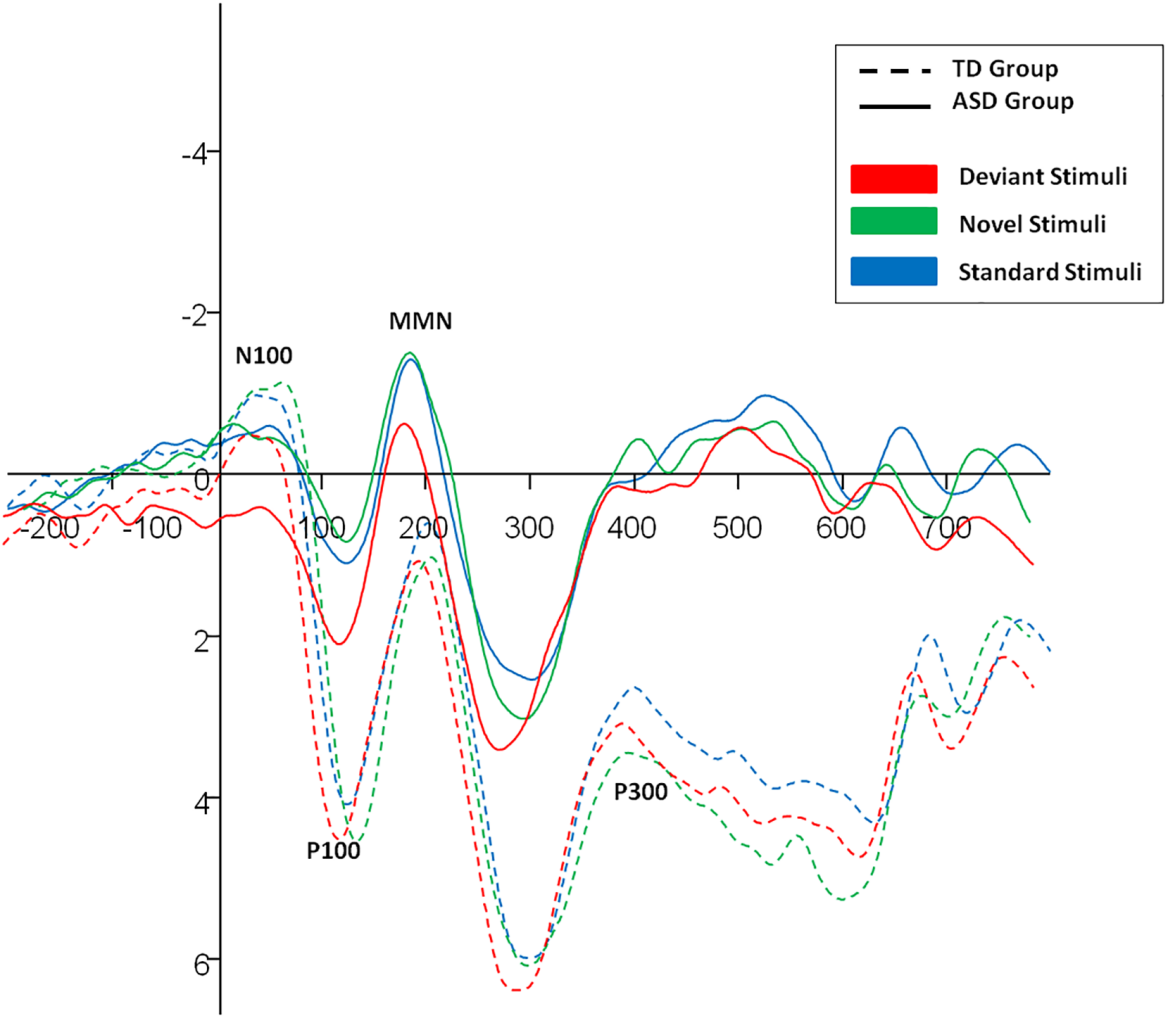
Average P100, N100, MMN and P300 ERPs in response to three image types in the ASD and TD groups.

Table 1 summarizes ASD and TD means and standard deviations for each ERP component of interest in addition to all main effects and interactions for Group. For P100 latencies, the significant main effect for Group [eta = .38] indicated ASD children had slower P100 latencies on average than TD children did. There were no significant interactions between Group and other independent variables regarding P100 latencies (see Table 1). For P100 amplitudes, the main effect for Group was not significant but there was a significant Group x Hemisphere interaction [p = 0.007, eta = 0.95]; simple effects analyses of this interaction indicated the ASD group displayed marginally smaller P100 amplitudes than TD children did in the right hemisphere [ASD: M = 2.44 (SD = 3.15) vs. TD: M = 4.53 (SD = 5.12), t = -2.375, p = 0.020) but there was no ASD group difference for the left hemisphere (ASD: M = 2.29 (SD = 3.01) vs. TD: M = 3.33 (SD = 4.83), t = -1.240, p = 0.218) [see Fig 2].

**Table 1:** Effects of ASD Group and its interactions with image type, hemisphere, and electrode on ERPs during a visual oddball task.

ERP Component	*ASD Group*^1^	*TD Group*^1^	*Univariate Effect*			
	*M ± SE*	*M ± SE*	*ASD Group*	*ASD Group x Image Type*	*ASD Group x Hemisphere*	*ASD Group x Electrode*
P100 latencies	1.31±3.70	1.04±2.97	30.01**	0.15	0.84	0.85
P100 amplitudes	2.37±0.32	3.93±0.49	6.37	0.01	7.57*	2.69
N100 latencies	62.97±1.48	59.49±1.48	2.63	0.10	5.33	0.95
N100 amplitudes	-0.02±0.12	-0.73±0.09	22.12**	0.07	1.47	3.41
P300 latencies	3.90±8.34	4.05±8.13	1.63	0.18	2.13	0.05
P300 amplitudes	-1.89± 0.38	0.55±0.57	11.34**	0.03	1.68	3.59
MMN latencies	2.41±4.42	2.32±3.33	2.70	0.003	0.97	0.94
MMN amplitudes	2.99±0.46	5.25±0.70	6.44	0.002	4.25	3.34
N400 latencies	-3.08±0.38	-2.70±0.73	0.19	1.04	0.02	2.68
N400 amplitudes	4.30±4.90	4.18±5.11	2.62	0.01	7.15*	2.80

^1^: ASD Group = Autistic Spectrum Disorder Group; TD Group = Typically Developing Group.

* p < 0.01

** p < 0.001.

### N100 component

Regarding N100 latencies, the main effect for Group and its interactions with all other independent variables were not significant (all p’s > .01) [see Table 1]. However, for N100 amplitudes, the significant effect for Group [p < .001, eta = .33] indicated children with ASD displayed weaker N1 amplitudes than did TD children during exposure to images. There were no significant interactions between Group and other independent variables regarding N100 amplitudes, though the effect for ASD Group x Hemisphere approached significance [p = 0.023,eta = 0.60].

### P300 and MMN components

No significant main effects or interactions for ASD group emerged for P300 latencies However, for P300 amplitudes, the main effect for Group [p = 0.001, eta = .24] indicated children with ASD displayed larger P300 amplitudes than did TD children during exposure to images. No significant interactions were found between ASD group and other independent variables. As predicted, no Group main effects or interactions were observed for the MMN component (see Table 1).

### N400 components

Regarding N400 latencies, neither the main effect for Group nor its interactions with other independent measures were significant (Table 1). For N400 amplitudes, Table 1 also indicates the main effect for ASD group was not significant but there was one significant interaction for Group x Hemisphere [p< .01, eta = 0.59]. However, simple effects analyses of this interaction did not identify significant ASD group differences in N400 amplitudes within either hemisphere (p’s > .01).

## Discussion

This study evaluated differences in ERP responses between children with ASD and TD children during the course of information processing within a visual oddball paradigm. Hypotheses were partially supported. Specifically, as expected, children with ASD displayed ERPs reflecting possible deficits in initial orienting and allocation of attention resources including slower P100 latencies, marginally weaker P100 amplitudes in the right hemisphere, and significantly weaker N100 amplitudes. Although the hypothesized absence of group differences regarding the MMN was also supported, children with ASD displayed comparatively stronger P300 amplitudes during the task, contrary to predictions. Finally, although a significant overall effect emerged for the N400 x Hemisphere interaction, simple effects analyses failed to isolate statistically significant group differences in either hemisphere. Possible implications of each main finding are discussed briefly below.

Past ERPs research has linked P100 and N100 components, respectively to initial attention resource expenditures and orienting of attention [34–36]. The pattern of associated results suggested that children with ASD were initially slower in allocating attentional resources to the task and orienting initial attention towards visual stimuli. Reassuringly, these findings align with initial predictions, conclusions of previous reviews [37] and past evidence linking ASD to early ERPs abnormalities in occipital-parietal areas [19,20] in addition to research indicating people with ASD experience impairments in the fusiform gyrus, a region involved in the generating posterior-visual P100 and N100 responses from the lateral extrastriate cortex [34,38–40]. Although structural causes of these deficits cannot be determined from the present data, they do bolster evidence for neural markers of early attention deficits within the visual modality among children with ASD. Notably, these ERP patterns did not vary as a function of stimulus deviance or novelty in the oddball task. As such, deficits in early visual attention of children with ASD may be general and linked to initial stimulus onsets rather than specific and associated with novel or deviant stimulus onsets.

Finally, with respect to early ERP components, ASD children displayed comparatively weaker P100 amplitudes localized in the right hemisphere. The associated effect sized was large (eta = .95) but only approached statistical significance (p = 0.02). As such, the implications of this finding warrant consideration in extensions with larger samples testing the hypothesis that initial deficits in visual attention allocation of ASD children may be more prominent in right hemisphere which can have comparatively more involvement in visual-spatial perception and processing of emotion.

In later stages of visual processing, children with ASD displayed significantly larger P300 amplitude during exposure to visual stimuli relative to TD children. The P300 has been used as a neural marker reflecting sustained attention to target stimuli, alertness, and increased allocation of attention resources to the task at hand [7,21–23,27,28]. Past research had suggested groups with ASD and TD groups do not generally differ on the P300 [29,30] so larger amplitudes among children with ASD in this study were not expected. Conservatively, this finding indicates children with ASD are able to sustain attention on briefly presented standard, deviant, and novel visual stimuli at least as well as TD children can. Although we had predicted ASD children might display ERPs reflecting differential processing strategies at a later stage (i.e., N400), it is possible that comparatively larger P300 amplitudes in this group reflected an alternate compensatory strategy reflecting sustained attention to visual stimuli following deficient neural responses experienced shortly after stimulus onsets. Future work is warranted to evaluate this conjecture.

MMN results were in line with initial predictions, as ASD and TD groups did not differ on this component. As such, relatively automatic change detection processes may have been intact among children with ASD compared to controls, at least in relation to a passive viewing task that required no formal response execution from the children. This finding is at odds with past research identifying MMN effects among children with ASD in contexts having higher task demands [32]. Nonetheless, our results unambiguously challenge contentions from related work that children with ASD have “general” deficits in change detection processing [8,32].

Finally, the hypothesis that groups would show differences in N400 responses to visual images that reflect preferences for imagery versus semantic strategies in processing was not resolved from the pattern of results. Although a significant Group x Hemisphere interaction was observed for central-frontal N400 amplitudes, simple effects analyses of group differences within each hemisphere were not significant. Past work indicates the N400 is sensitive to conceptual features and semantic context of stimuli [41,42] that could reflect automatic semantic priming of stimuli [43]. We assumed that groups might show differences in strategies related to processing images based on differential use of imagery versus sematic processing strategies. The absence of N400 differences in simple effects analyses suggested children with ASD and TD children do not differ, at least dramatically, in their manner of processing of images at this stage.

In summary, this research bolstered evidence for possible deficits in initial allocation and orienting of attention to visual stimuli based on early ERPs elicited among children with ASD compared to controls. Building upon previously reported P100 and N100 effects [37], this research indicated such affects extend to pre-adolescent children, broader than previously used visual stimulus arrays, and groups in an understudied cultural context (i.e., China). Conversely, however, children with ASD displayed larger P3 amplitudes than controls did across stimulus categories, suggesting relative strengths related to alertness and sustained attention as stimulus presentations persisted. Finally, the absence of differences between children with ASD and controls on MMN and N400 components, respectively, suggest that neural markers related to the detection of novel stimulus events and use of semantic versus imagery-based strategies for handling visual stimuli may be more similar than different between these groups.

Its main implications aside, this study has several limitations that should be discussed as foundations for future extensions. First, the overall sample size was comparable to that of other ERP studies on autism but was also relatively small. Coupled with the use of a more stringent than conventional significance cutoff to reduce the risk for Type I errors, the small N ensured that any detected effects had medium or large effect sizes. Extensions with larger samples may provide more statistical power to detect subtle ERP differences between these groups or interactions with other factors.

Second, although groups did not differ in age, the ASD group had a wider age range than the TD group did. Fortunately, comprehension and recognition of the stimulus materials was not affected by age or ASD status and results were similar after dropping the youngest or older member of the ASD group from analyses. Nonetheless, extensions might focus upon and compare narrower age bands to clarify possible developmental differences. Third, because results may not generalize beyond the current paradigm (i.e., passive viewing in a visual oddball task), extensions are warranted to tasks requiring active responses to visual stimuli (e.g., tasks requiring speed or decision-making). On a related note, inconclusive N400 results point to the need for follow-up studies that evaluate use of semantic versus imagery-based strategies in processing standard versus novel information.

## References

[1] American Psychiatric Association. Diagnostic and statistical manual of mental disorders (DSM-5®). American Psychiatric Pub, 2013.

[2] McEvoy RE, Rogers SJ, Pennington BF. Executive function and social communication deficits in young autistic children. J Child Psychol. Psychiat1993 May;34(4): 563–578. 10.1111/j.1469-7610.1993.tb01036.x 7685360

[3] Lopez BR, Lincoln AJ, Ozonoff S, Lai Z. Examining the relationship between executive functions and restricted, repetitive symptoms of autistic disorder. J Autism Develop Dis. 2005 8; 35(4): 445–460. 10.1007/s10803-005-5035-x 16134030

[4] Ceponiene R, Lepisto T, Shestakova A, Vanhala R, Alku P, Naatanen R. Speech-sound-selective auditory impairment in children with autism: They can perceive but do not attend. Proceed Nat Acad Sciences, 2003 4 29; 100(9): 5567–5572. 10.1073/pnas.0835631100 PMC154385 12702776

[5] Näätänen R. The mismatch negativity: a powerful tool for cognitive neuroscience. Ear Hear.1995;16(1): 6–18. 7774770

[6] Garrido MI, Kilner JM, Stephan KE, Friston KJ. The mismatch negativity: a review of underlying mechanisms. Clin. Neurophysiol. 2009 3; 120(3): 453–463. doi: 10.1016/j.clinph.2008.11.029 19181570 10.1016/j.clinph.2008.11.029 PMC2671031

[7] Sokhadze E, Baruth J, Tasman A, Sears L, Mathai G, El-Baz A, et al Event-related potential study of novelty processing abnormalities in autism. Appl Psychophysiol Biofeedback. 2009 2 06;34(1): 37–51. doi: 10.1007/s10484-009-9074-5 19199028 10.1007/s10484-009-9074-5

[8] Clery H, Bonnet-Brilhault F, Lenoir P, Barthelemy C, Brueau N, Gomot M. Atypical visual change processing in children with autism: An electrophysiological study. Psychophys. 2013 1 14; 50(3): 240–252. 10.1111/psyp.12006 23316882

[9] Herrmann CS, and Knight RT. Mechanisms of human attention: Event related potentials and oscillations. Neurosc Biobehav Rev. 2001 8; 25(6): 465–476. 10.1016/s0149-7634(01)00027-6 11595268

[10] Gomot M, Blanc R, Cléry H, Roux S, Barthelemy C, Bruneau N. Candidate electrophysiological endophenotypes of hyperreactivity to change in autism. J Autism Developl Diss.2011 6;41(6): 705–714. 10.1007/s10803-010-1091-y 20827502

[11] Boyd BA, Baranek GT, Sideris J, Poe MD., Watson LR., Patten E. Sensory features and repetitive behaviors in children with autism and developmental delays. Autism Res. 2010 4 05;3(2): 78–87. PMCID: PMC3071028 doi: 10.1002/aur.124 20437603 10.1002/aur.124 PMC3071028

[12] Gomot M, Wicker B. A challenging, unpredictable world for people with Autism Spectrum Disorder. Internat J Psychophys.2012 2; 83(2): 240–247. 10.1016/j.ijpsycho.2011.09.017 21968196

[13] Villalobos ME, Mizuno A, Dahl BC, Kemmotsu N, Muller RA. Reduced functional connectivity between V1 and inferior frontal cortex associated with visuomotor performance in autism. NeuroImage. 2005 4 15; 25(3): 916–925. doi: 10.1016/j.neuroimage.2004.12.022 15808991 10.1016/j.neuroimage.2004.12.022 PMC3319340

[14] Welchew DE, Ashwin C, Berkouk K, Salvador R, Suckling J, Baron-Cohen S, et al Functional disconnectivity of the medial temporal lobe in Asperger’s syndrome. Biol Psychiatry. 2005 5 1;57(9): 991–998. doi: 10.1016/j.biopsych.2005.01.028 15860339 10.1016/j.biopsych.2005.01.028

[15] Coles MGH, Rugg MD. Event-related brain potentials: an introduction In: Rugg MD, Coles MGH (eds) Electrophysiology of mind: event-related brain potentials and cognition. Oxford: Oxford University Press; 1995 pp 40–85.

[16] Luck SJ, Hillyard SA, Mouloua M, Woldorff MG,;Clark VP., Hawkins HL. Effect of spatial cueing on luminance detectability: Psychophysical and electrophysiological evidence for early selection. Journal of Experi Psychol: Hum Percep Perform. 1994 8,20(4): 887–904. 10.1037//0096-1523.20.4.887 8083642

[17] Heinze HJ, Luck SJ, Mangun GR,. Visual event-related potentials index focused attention within bilateral stimulus arrays. I. Evidence for early selection. Electroencephalography and clinical neurophysiology. 1990 6, 75(6): 511–527. 1693896 10.1016/0013-4694(90)90138-a

[18] Gomez-Gonzales CM, Clark VP, Fan S, Luck S, Hillyard SA. Sources of attention-sensitive visual event-related potentials. Brain topography. 1994 9,7(1): 41–51. 7803199 10.1007/BF01184836

[19] Hopf JM, Vogel E, Woodman G, Heinze HJ, Luck SJ. Localizing visual discrimination processes in time and space, J. Neurophysiol. 2002 10; 88(4): 2088–2095. 12364530 10.1152/jn.2002.88.4.2088

[20] Yamazaki T, Kamijo K, Kenmochi A, Fukuzumi S, Kiyuna T, Kuroiwa Y. Multiple equivalent current dipole source localization of visual event-related potentials during oddball paradigm with motor response. Brain Topogr. 2000 3;12(3): 159–175. 10791680 10.1023/a:1023467806268

[21] Azizian A, Polich J. Evidence for attentional gradient in the serial position memory curve from event-related potentials. J Cog Neurosc. 2007 12; 19(12): 2071–2081. 10.1162/jocn.2007.19.12.2071 PMC2748728 17892393

[22] Mecklinger A, Pfeifer E. Event-related potentials reveal topographical and temporal distinct neuronal activation patterns for spatial and object working memory. Cog Brain Res. 1996 10;4(3): 211–224. 10.1016/s0926-6410(96)00034-1 8924049

[23] Celesia GG, Brigell M. Event-related potentials. Curre Opin Neurol. 1992 10;5(5): 733–739. 1392146

[24] Soltani M, Knight RT. Neural origins of the P300. Crit Rev Neurobiol. 2000, 14(3–4): 199–224. 12645958

[25] Frodl T, Hampel H, Juckel G, Burger K, Padberg F, Engel RR, et al l. Value of event-related P300 subcomponents in the clinical diagnosis of mild cognitive impairment and Alzheimer's disease. Psychophys. 2002 3;39(2): 175–181. 10.1017/S0048577202010260 12212666

[26] Karayanidis F, Andrews S, Ward PB, Michie PT. ERP indices of auditory selective attention in aging and Parkinson's disease. Psychophysi. 1995 7; 32(4): 335–350. 10.1111/j.1469-8986.1995.tb01216.x 7652110

[27] Friedman D, Simpson GV, Hamberger M. Age-related changes in scalp topography to novel and target stimuli. Psychophys.1993 7; 30(4): 383–396. 10.1111/j.1469-8986.1993.tb02060.x 8327624

[28] Knight RT. Decreased response to novel stimuli after prefrontal lesions in man. Electroencepha Clin Neurophys. 1984 2;59(1): 9–20. 10.1016/0168-5597(84)90016-9 6198170

[29] Larson MJ, South M, Krauskopf E, Clawson A, Crowley MJ. Feedback and reward processing in high-functioning autism. Psychiat Res.2011 5 15;187(1–2): 198–203. 10.1016/j.psychres.2010.11.006 21122921

[30] Sokhadze EM, Baruth JM, Sears L, Sokhadze GE, El-Baz AS, Williams E, et al Event-related potential study of attention regulation during illusory figure categorization task in ADHD, autism spectrum disorder, and typical children. J Neurother. 2012 3 2;16(1): 12–31. doi: 10.1080/10874208.2012.650119 23329879 10.1080/10874208.2012.650119 PMC3544080

[31] Jahshan C Cadenhead KS, Rissling AJ,. Automatic sensory information processing abnormalities across the illness course of schizophrenia. Psychol Med. 2012 1; 42(01): 85–97. 21740622 10.1017/S0033291711001061 PMC3193558

[32] Cléry H., Roux S, Houy-Durand E, Bonnet-Brilhault F, Bruneau N, Gomot M. Electrophysiological evidence of atypical visual change detection in adults with autism. Front Human Neurosc.2013 3 06;7(62): 1–11. PMCID: PMC3589704 10.3389/fnhum.2013.00062 PMC3589704 23507615

[33] Whitehouse AJO, Maybery MT, Durkin K. Inner speech impairments in autism. J Child Psych Psychiat. 2006 8 1; 47(8): 857–865. 10.1111/j.1469-7610.2006.01624.x 16899000

[34] Hillyard SA, Anllo-Vento L. Event-related brain potentials in the study of visual selective attention. Proceed Nat Acad Sciences, 1998, 95(3): 781–787. PMCID:PMC33798 10.1073/pnas.95.3.781 PMC33798 9448241

[35] Näätänen R, Michie PT. Early selective-attention effects on the evoked potential: a critical review and reinterpretation. Biol Psychol. 1979 3;8(2): 81–136. 465623 10.1016/0301-0511(79)90053-x

[36] Luck SJ, Heinze HJ, Mangun GR, Hillyard SA. Visual event-related potentials index focused attention within bilateral stimulus arrays, II: functional dissociation of P1 and N1 components. Electroencephalogr Clin Neurophysiol.1990 6;75(6): 528–542. 1693897 10.1016/0013-4694(90)90139-b

[37] Jeste SS, Nelson CA. Event related potentials in the understanding of autism spectrum disorders: an analytical review. JAutism and Develop Diss. 2009 3 1;39(3): 495. 10.1007/s10803-008-0652-9 PMC4422389 18850262

[38] Hall GB, Doyle KA, Goldberg J, West D, Szatmari P. Amygdala engagement in response to subthreshold presentations of anxious face stimuli in adults with autism spectrum disorders: preliminary insights. PLoS ONE. 2010 5 25;5(5): e10804 PMCID: PMC2876036 doi: 10.1371/journal.pone.0010804 20520836 10.1371/journal.pone.0010804 PMC2876036

[39] Dalton KM, Nacewicz BM, Johnstone T, Schaefer HS, Gernsbacher MA, Goldsmith HH, et al Gaze fixation and the neural circuitry of face processing in autism. Nat Neurosci. 2005 3 06; 8(4): 519–526. PMCID: PMC4337787 doi: 10.1038/nn1421 15750588 10.1038/nn1421 PMC4337787

[40] Marsh LE, Hamilton AF. Dissociation of mirroring and mentalising systems in autism. Neuroimage. 2011 6 1;56(3): 1511–1519. doi: 10.1016/j.neuroimage.2011.02.003 21310248 10.1016/j.neuroimage.2011.02.003

[41] Barrett SE, Rugg MD. Event-related potentials and the semantic matching of pictures. Brain Cogn. 1990 11;14(2): 201–212. 2285513 10.1016/0278-2626(90)90029-n

[42] McPherson WB, Holcomb PJ. An electrophysiological investigation of semantic priming with pictures of real objects. Psychophys. 1999 1; 36(1): 53–65. 10.1017/s0048577299971196 10098380

[43] Blackford T, Holcomb PJ, Grainger J, Kuperberg GR. A funny thing happened on the way to articulation: N400 attenuation despite behavioral interference in picture naming. Cogn. 2012 4; 123(1): 84–99. PMCID: PMC3634574 10.1016/j.cognition.2011.12.007 PMC3634574 22245030

